# The Peripheral Immune Landscape of Breast Cancer: Clinical Findings and In Vitro Models for Biomarker Discovery

**DOI:** 10.3390/cancers13061305

**Published:** 2021-03-15

**Authors:** Sofia Batalha, Sofia Ferreira, Catarina Brito

**Affiliations:** 1 Instituto de Biologia Experimental e Tecnológica (iBET), Apartado 12, 2781-901 Oeiras, Portugal; sofia.batalha@ibet.pt; 2 Instituto de Tecnologia Química e Biológica António Xavier, University Nova de Lisboa, Avenida da República, 2780-157 Oeiras, Portugal; 3 Instituto Português de Oncologia de Lisboa Francisco Gentil, Rua Prof Lima Basto, 1099-023 Lisboa, Portugal; asferreira@ipolisboa.min-saude.pt

**Keywords:** breast cancer, biomarker, blood, T cell, NK cell, macrophage, MDSC, 3D cell models, immunosuppression, patient data

## Abstract

**Simple Summary:**

Breast cancer is a major health concern as it remains the deadliest for women worldwide. As for other cancer types, the immune system is determinant for how the body fights the tumor and responds to therapy. Better medical care and therapy assignment can thus be obtained by a deeper understanding of the immune state of breast cancer patients and how it changes with disease and treatment. Such information becomes more relevant and accessible if collected easily from a blood test. This review summarizes current knowledge on breast cancer patients’ immune profile obtained from peripheral blood and serves as a starting point for further investigation. Finally, we discuss the latest advances and current challenges in experimental models to study the interactions between human cancer and immune cells, with the intent of bridging the gap between patient immune profiling and functional and therapeutic significance.

**Abstract:**

Breast cancer is the deadliest female malignancy worldwide and, while much is known about phenotype and function of infiltrating immune cells, the same attention has not been paid to the peripheral immune compartment of breast cancer patients. To obtain faster, cheaper, and more precise monitoring of patients’ status, it is crucial to define and analyze circulating immune profiles. This review compiles and summarizes the disperse knowledge on the peripheral immune profile of breast cancer patients, how it departs from healthy individuals and how it changes with disease progression. We propose this data to be used as a starting point for validation of clinically relevant biomarkers of disease progression and therapy response, which warrants more thorough investigation in patient cohorts of specific breast cancer subtypes. Relevant clinical findings may also be explored experimentally using advanced 3D cellular models of human cancer–immune system interactions, which are under intensive development. We review the latest findings and discuss the strengths and limitations of such models, as well as the future perspectives. Together, the scientific advancement of peripheral biomarker discovery and cancer–immune crosstalk in breast cancer will be instrumental to uncover molecular mechanisms and putative biomarkers and drug targets in an all-human setting.

## 1. Introduction

Breast cancer remains the leading cause of cancer-related deaths for women worldwide. In 2018, 2.1 million women were diagnosed with breast cancer and 626,679 died from it [1]. Breast cancer is curable in approximately 70–80% of patients with early-stage disease [2], but, despite new available treatments, advanced/metastatic breast cancer remains an incurable disease with a median overall survival of about three years and a five-year survival rate of around 25% [3].

Breast cancer is a heterogeneous disease, which can be classified according to histology and molecular characteristics. The most common invasive histological subtypes are ductal carcinoma, currently named as “no special type”, and lobular carcinoma. Based on gene expression signature (PAM50), breast tumors can also be divided in intrinsic subtypes: Luminal A, Luminal B, HER2-enriched, Normal-like, and Basal-like [4]. Both histological and molecular features have an impact on prognosis and treatment. In daily clinical practice, surrogate intrinsic subtypes are used. This classification takes in account the histology, the receptor expression, and the proliferation index. Thus, tumors can be classified as: Luminal A-like (high estrogen receptor (ER) and progesterone receptor (PR) expression, HER2 negative, low grade, and low Ki67 index), Luminal B-like HER2 negative (lower expression of ER and PR, HER2 negative, higher grade, and high Ki67 index), Luminal B-like HER2 positive (lower expression of ER and PR, HER2 positive, higher grade, and high Ki67 index), Non-luminal HER2-enriched (ER and PR negative, HER2 positive, high grade, and high Ki67 index), and Triple-negative (ER, PR and HER2 negative, high grade, and high Ki67 index) [2].

In the early setting, with curative intent, Luminal A-like tumors are usually treated with surgery followed by radiotherapy and endocrine therapy. Chemotherapy is usually less used, except for large tumors or nodal involvement. Luminal B-like tumors can be treated with (neo)adjuvant chemotherapy followed by surgery, radiotherapy, and endocrine therapy. HER2 positive tumors are treated with (neo)adjuvant chemotherapy combined with anti-HER2 agents followed by surgery and radiotherapy. Early triple negative tumors are treated with (neo)adjuvant chemotherapy followed by surgery and radiotherapy [5]. In the advanced setting, endocrine therapy and CDK4/6 inhibitors play a crucial role as first lines of treatment of Luminal-like tumors and anti-HER2 agents are central in the treatment of HER2 positive tumors, while immune checkpoint inhibitors combined with chemotherapy can be used in triple negative tumors [6].

The clinical outcome of breast cancer patients is not only dependent on the therapeutic action of drug treatments. The immune system is known to play a major role in all stages of cancer development, initially in the elimination of the first malignant cells and then progressively becoming subverted by the tumor to promote cancer growth and metastization [7]. In these interactions, cells from both the lymphoid (T cells, B cells, and natural killer (NK) cells) and the myeloid (monocytes/macrophages, and dendritic cells (DC), neutrophils, myeloid-derived suppressor cells (MDSC)) lineages are heavily implicated. T cells are a very heterogeneous population: on one hand, CD8^+^, CD4^+^, and γδT cells are directly involved in the specific killing of tumor cells, directed by the recognition of tumor antigens. On the other hand, regulatory T cells (T_Regs_) promote the establishment of a pro-tumoral environment [7,8,9]. NK cells are also able to induce tumor cell killing based on the detection of activating danger signals on the tumor cell surface, while also secreting pro-inflammatory cytokines [7,8,9]. B cells are of major importance for the establishment of anti-tumoral immunity due to their role in antibody production and in the formation of tertiary lymphoid structures in the periphery of the primary tumor, where they also exert the function of presenting antigen to T cells, activating them [7,8,9]. This role of antigen presentation is also performed by dendritic cells, which hold the unique ability to cross-present tumor antigens to CD8^+^ T cells, promoting direct cell killing [7,8,9]. Monocytes and macrophages hold a dual role in cancer progression given their functional plasticity and the ability to become either tumor-promoting or anti-tumorigenic. Depending on the microenvironmental and systemic cues, macrophages may synergize with CD8^+^ and NK cell action by killing tumor cells and secreting pro-inflammatory cytokines; more often, they acquire a pro-tumoral phenotype and promote a strong immunosuppressive immune milieu, which leads to cancer growth and metastization [7,8,9]. Neutrophils may also be polarized into a tumor-promoting phenotype, and are currently regarded as being directly involved in metastasis formation [7,8,9]. Other suppressive cells in the cancer context include the MDSC, a group of immature myeloid cells of the monocytic and granulocytic lineages with the capacity to subvert the anti-tumor function of other immune populations [7].

This influence of the immune system upon cancer development has been studied for a long time. In the mid-20th century, Burnet and Thomas postulated the theory of immunosurveillance based on studies using tumor transplantation models [10]. Although the original formulation raised controversy, it inspired numerous studies along the following decades that contributed to a better understanding of the immune system as a key player in tumor development. Dunn et al., 2002 proposed the concept of immunoediting, based on three “E”s—elimination, equilibrium, and escape—and recognized both positive and negative effects of the host–tumor interaction [11]. Later, the involvement of the immune system in the treatment of cancer was also investigated, after several studies showed that conventional chemotherapy effects are also mediated by immunogenic actions [12]. This antitumor immune activation was identified as one of the mechanisms responsible for metronomic (low dose continuous) chemotherapy efficacy [13], with recent reports indicating that the immunogenic cell death induced by anthracycline or taxane treatment also subverts the immunosuppressive environment at the tumor site [14]. Regarding targeted agents, the anti-HER2 antibody trastuzumab may also be considered an immune modulatory agent, due to its ability to induce antibody-dependent cell-mediated cytotoxicity (ADCC) and influence the activity of NK and T cells, macrophages, and dendritic cells [15,16]. Similarly, the two other approved HER2-targeting agents pertuzumab and T-DM1 were also shown to promote immune cell engagement via ADCC [17,18].

The application of immunotherapies to breast cancer became more prominent in recent years, following the trend of other malignancies [19]. In this regard, and considering the currently available immune-targeted drugs, checkpoint inhibitors have already showed benefit in the triple negative breast cancer (TNBC) context. In the Impassion 130 trial, the anti-PD-L1 antibody atezolizumab was used in combination with nab-paclitaxel to treat patients with PD-L1+ advanced TNBC, resulting in a significant clinical benefit not only for progression-free survival (PFS) but also for overall survival (OS) [20]. More recently, data from early phase studies demonstrated that the association of pembrolizumab with neoadjuvant chemotherapy for high-risk early TNBC patients was feasible and had a promising antitumor activity. An exploratory analysis showed a correlation between the occurrence of pathological complete response (pCR) and the expression of tumor PD-L1 and the infiltration by tumor infiltrating lymphocytes (TILs) [21]. In fact, the role of TILs as independent predictors of response to neoadjuvant chemotherapy has been previously validated in patients [22]. In parallel, other trials are ongoing regarding the use of anti-CTLA-4 agents and regimens combining immune checkpoint blockade (ICB) with several targeted therapies [19,23].

Concurrent with the evidence that immunotherapies are beneficial in breast cancer treatment, years of studies with thousands of patients have consistently showed that for early stage HER2+ and TNBC cases the presence and increased numbers of TIL are associated with better disease prognosis and response to therapy [24]. For this reason, it has been proposed to incorporate TIL analysis as predictive and prognostic biomarkers in clinical practice. However, as TIL are by definition contained within the tumor mass, analysis is always dependent on invasive biopsies or surgical resection. Therefore, TIL evaluation is not possible for every patient and it becomes difficult to evaluate patients’ progression at frequent intervals. On the other hand, the use of peripheral blood immune biomarkers would be especially useful to evaluate disease progression and treatment response, allowing for faster, cheaper, and more precise monitoring of patients’ status—and, ideally, for tailoring treatments. Assessing features of systemic immunity would also prove useful in metastatic disease as it would reflect not only events of the primary tumor but also of distant metastasis. Several studies failed in the identification of predictive biomarkers, even in tumor types where immunotherapy had a huge impact [25]. Thus, the area of immune blood biomarkers in solid tumor patients remains underdeveloped and so far there is no validated predictive biomarker for breast cancer. In fact, concise information on the peripheral immune status of breast cancer patients of all stages is still dispersed and often neglected.

This review aims to cover the most relevant immune parameters and biomarkers with current potential to be used as predictive and prognostic tools for breast cancer assessment in the clinic, as well as to further understand the immune mechanisms connected with tumor progression or regression. Finally, we discuss the existing cellular models applicable to explore the mechanisms behind these relationships of peripheral immune state and disease progression, their strengths, and future developments.

## 2. Systemic Immune Status

### 2.1. Peripheral Lymphocyte Count

The earliest [26] and easiest way to assess patients’ immune capacity has been through the absolute count of peripheral blood lymphocytes (PBL) or mononuclear cells (PBMC, which encompass all white blood cells minus granulocytes). In this regard, research across all breast cancer subtypes and disease stages has consistently showed that higher lymphocyte count at baseline, prior to therapeutic intervention, is an indicator of better prognosis (Table 1) [26,27,28,29,30,31]. In primary breast cancer patients undergoing neoadjuvant chemotherapy (NAC), lower absolute lymphocyte numbers were predictive of a shorter disease-free survival (DFS) and overall survival [27] and, conversely, higher lymphocyte counts were associated with a higher rate of pCR after NAC and tumor resection [28]. This trend was also observed in a cohort of elderly (over 65 years old) patients, where higher lymphocyte counts were positively associated with a higher three-year DFS both in univariate and multivariate analysis [29]. Additionally, in a subtype-restricted analysis, TNBC [31] and hormone receptor positive (HR^+^) [30] patients with baseline lymphocyte counts above a cut-off value were found to have significantly longer OS and DFS. On the other hand, no significant associations were found between lymphocyte number and disease prognosis in HER2^+^ breast cancer patients [30], even though HER2-overexpressing disease may be associated with a higher baseline PBL count [27]. A positive response to NAC was also found more frequently among patients who maintained a consistent level of PBL after the first cycle of treatment [27], already suggesting the potential of chemotherapy regimens to influence the anti-tumor immune response. It is worth noticing, however, that such promising results were observed in cohorts of primary breast cancer patients only, suggesting that metastatic disease requires additional readouts.

In recent years, other indexes combining counts of different immune cells with inflammatory potential have gained popularity in clinical oncology as indicators of the systemic immune status. Parameters such as the lymphocyte-to-monocyte (LMR), neutrophil-to-lymphocyte (NLR), and platelet-to-lymphocyte (PLR) ratios, among others, were shown to have a significant prognostic and predictive power in several solid tumors [40,41,42,43]. In breast cancer, NLR and LMR are highlighted in several studies as the indices displaying stronger correlation with disease prognosis (Table 1) [28,29,30,31,32,33,34,35,36,37,38]. In two distinct cohorts, Azab et al. categorized patients in four quartiles according to their baseline measures of NLR and observed a significant increase in short- and long-term mortality rate for patients in the highest NLR quartile [33,34], which corresponded to a 4 to 6 times higher probability of death at five years, depending on the cohort and comparing to the three lowest quartiles. In early-stage breast cancer patients undergoing NAC, NLR was also associated with prognosis. Patients presenting lower NLR values displayed longer DFS and OS [29,32], and had a higher probability of achieving a pCR after NAC [28,29,30]. This observation was especially marked for the TNBC subtype [29], in which low NLR values were associated with an average of 5.5 years longer OS; accordingly, higher NLR values were linked to higher mortality rates [35]. These results are not surprising considering that patients with elevated values of this index often present other indicators of worse prognosis such as higher levels of metastization, hormone receptor negativity, and HER2 positivity [35].

LMR evaluates the ratio between the absolute counts of lymphocytes and monocytes in circulation, and recent research has shown its potential as a prognostic guide in breast cancer [30,31,32,37,38]. Patients with a high LMR display longer DFS [30,32,37] and OS [30,32] than those with lower ratios, including cohorts of luminal [38] and triple-negative [31] breast cancer patients. Higher LMR values were also found more often in patients with less advanced tumor stages [31], hormone receptor positivity [37], and higher prevalence of CD8^+^ TIL in the tumor microenvironment (TME) [30], although other studies found these associations not significant [29,32]. This heterogeneity may be due to the lack of consensus in the cut-off values chosen for each index, which for LMR was found to range between 2.94 and 5.46 (almost a twofold difference) across studies. Of note, only one group was found to investigate the influence of different cut-off values on the final statistic [35].

The usefulness of the ratio of PLR is still a matter of debate, as literature is scarce and inconsistent (Table 1). While some authors claim there is no association between PLR and breast cancer prognosis [29,30], others present evidence for a significant capacity to predict patient’s survival [34,35,39]. In a group of over 700 breast cancer patients, high PLR was identified as an independent prognostic factor that predicted worse OS and the appearance of distant metastasis [39], with subtype-specific analysis revealing this association to be specific of Luminal B and Basal-like breast cancer. In fact, for the Luminal B subtype, PLR proved to be superior to age and T status in predicting cancer-specific survival. Similarly, patients in the highest PLR categories displayed larger tumors, higher NLR and metastasis rate and were more likely to be HER2^+^ [34,35]. Their mortality risk at five years was also significantly increased [35] and considered an independent predictive factor in a multivariate analysis, although with inferior capacity compared with NLR [34]. It is interesting to note that platelets seem to have a relevant role in cancer development, as they have been found to increase the local concentrations of VEGF, promoting angiogenesis, and to aggregate with tumor cells, improving their survival in the peripheral blood [34]. One of the reasons for the discrepancies reported may lie once again in the disparity of values chosen for the threshold of low vs. high PLR. In fact, when Koh et al. analyzed the sensitivity of their model to two already published cut-off values (185 [33,34] and 292 [39]), it became obvious that only one of them (185) returned a significant association in a multivariable analysis adjusted for confounders [35]. Given that each patient cohort is different, and that immune ratio cut-off values are often calculated based on receiver operator characteristic (ROC) curve analysis for distinct parameters (like DFS or OS), it is understandable that varying patient populations and prognosis indicators will result in distinct cut-off values. Therefore, it is vital to find a standardized range of acceptable threshold values to obtain more concise and comparable results that can be applied to clinical decision making.

Surprisingly, only one study was found to focus specifically on metastatic patients [36]. These appeared to follow a similar trend to non-metastatic cases, with low NLR and PLR and high LMR providing a survival advantage. Additionally, subtype stratification indicated high LMR as a positive prognostic factor for TNBC and HER2, with the latter benefiting also from a low PLR. In this study, no specific correlation was found between ER^+^ breast cancer prognosis and immune cell ratio indices.

The diversity of patient populations covered in the literature cited above—in terms of disease staging, subtype, treatment regimen, ethnicity, or sample size—is a favorable factor, increasing reliability of the results obtained. Nevertheless, more research is needed to validate the observations obtained from small cohorts, such as the HER2^+^ or metastatic breast cancer patients, which are usually much smaller than the others. It is also worth noting that most of these studies focus on disease prognosis as the main endpoint/readout, and that this may change throughout the duration of treatment and depending on the therapeutic regimen. It is then essential to complement these measures of prognosis with other readouts assessing response to therapy in the same or similar patient populations; this would facilitate the distinction of (potential) biomarkers that may interact with the treatment applied in each case.

Still, taking together these observations, there are already strong indicators that lymphocytes, monocytes, and neutrophils play a decisive role in shaping the fight against cancer at the organism level, as also highlighted recently in a review of the latest developments in the field [44].

### 2.2. Lymphocyte Function and Soluble Factors

The general immune state of breast cancer patients may also be gauged by the levels of immune modulatory factors found in plasma and by the functional state of immune cells per se. As reported for other cancer types [45,46,47], circulating immune cells of breast cancer patients display reduced expression of pro-inflammatory markers [48,49,50,51,52,53] and are less amenable to stimulation [49,54,55], when compared with healthy donors. This can be seen by the reduced secretion of typical inflammatory cytokines such as IL-6, IL-12, IL-2, IFN-γ, TNF-α, IL-8, GM-CSF, and IL-1β [48,49,50] and the increase in production of IL-4, IL-10, and PGE_2_ [48], which are commonly considered drivers of a tumor-promoting microenvironment [56,57,58]. Consistently, Elashi et al. found that patients’ baseline levels of checkpoints TIM-3, TIGIT, and PD-L1 were not only higher than in healthy donors but also than in the tumor site [51]. This apparent lack of pro-inflammatory reactivity, seen also after stimulation with phorbol myristate acetate (PMA) [49], may be explained by a general fragility of circulating immune cells, which appear to have a higher degree of membrane integrity loss than those of healthy donors—as seen by a higher level of spontaneous lactate dehydrogenase (LDH) release, which becomes more pronounced with disease progression [55].

However, it is interesting to note that metastatic disease seems to invert to some extent the tendency for less reactive circulating immune cells. It has been reported that in metastatic patients there was increased production of TNF-α and IL-1β [49] compared with early breast cancer patients, and upregulation of CD14 and CD40 [53], two receptors necessary for macrophage pro-inflammatory function and antigen presentation. This effect may be subtype-dependent though, as another study reports that metastatic HER2^−^ patients exhibited reduced production of IL-2, IL-1β, IL-8, IL-6, and IL-10 compared with HER2^+^ cases [52]. A stage-dependent increase in the expression of the anti-inflammatory markers FOXP3 and PD-L1 was also found in one cohort of patients of all subtypes (predominantly Luminal and HER2^+^) [53]. The highest expression of PD-L1 and FOXP3 correlated with a higher production of IFN-γ and diminished secretion of TGF-β2, respectively.

Unfortunately, to date very few studies have investigated putative links between these markers of immune function and breast cancer prognosis or response to therapy. For metastatic patients, high levels of IL-6 may predict worse disease prognosis [59]. There is also evidence that after systemic therapy with taxane regimens there is an increased production of IL-2, IL-6, GM-CSF, and IFN-γ, while secretion of IL-1, TNF-α, and PGE_2_ declines [50]; however, it is still unknown whether this precludes an actual clinical benefit for the patient. For TNBC, Foulds et al. found a specific inflammation-related mRNA signature, consisting of high expression of CD163 (a scavenger receptor associated with M2 macrophage polarization [60]) and CXCR4 (a chemokine receptor involved in monocyte function and deployment to the tissue [61]) and low expression of THBS1 (an activator of the TGF-β pathway [62] and proposed breast cancer biomarker [63]), which correlated with improved relapse-free survival in a cohort of 186 patients [64].

## 3. The Lymphoid Lineage

At the interface of cancer development and immune response, lymphocytes have been the core of oncoimmunology research from its inception. The presence and phenotype of TIL are acknowledged as major influencers of disease progression or regression in solid tumors [24], with potent prognostic implications for several malignancies, especially in the case of the so called “hot tumors”. These tumor types are characterized by an increased rate of lymphocyte infiltration, which has been linked to greater mutational load and higher levels of genetic instability, known to lead to the generation of more neoantigens and consequent activation and recruitment of immune cells [23,25,65,66,67]. Traditionally, breast cancer was considered an instance of “cold tumor” given the generally low level of infiltration and its lower rate of somatic mutation compared with highly mutational cancers like non-small cell lung cancer or melanoma [23], where the application of immunotherapies has been a reality for several years [68]. However, reports from the last decade have shown that, when stratified by subtype, HER2^+^ and TNBC have sufficient genomic instability and proliferation rate to sustain lymphocyte infiltration, while HER2 itself becomes a TAA promoting immune activation [23,69,70]. Accordingly, the presence of elevated numbers of TIL in the tumor microenvironment is associated with better disease prognosis and predicts a positive response to chemotherapy in these subtypes, which are more likely to harbor TIL than Luminal A or B breast cancers [19,23,69,70]. The role of TIL in breast cancer progression and prognosis lies outside the scope of this work as it has been thoroughly discussed elsewhere [24]. Nonetheless, it demonstrates that T, B, and NK cell phenotype and function are fundamental indicators of how the immune system is fighting against cancer.

### 3.1. T Cells

**Figure 1:**
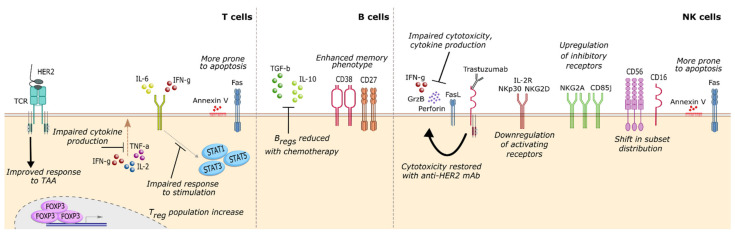
Surface markers and cytokine response of lymphoid peripheral immune cell populations associated with breast cancer occurrence and/or progression. In the lymphoid lineage, T cells and NK cells acquire a stronger suppressive phenotype in breast cancer patients including diminished response to, and production of, pro-inflammatory cytokines, higher susceptibility to apoptosis and impaired cytotoxic response—although the latter may be reversed upon exposure to tumor-associated antigens (T cells, via binding of HER2 to the TCR) or anti-HER2 therapeutic antibodies (NK cells, via binding of trastuzumab to CD16). B cells were also found to respond to chemotherapy regimens by reducing the B_reg_ population, thus promoting anti-tumor immune function. NK, natural killer cells; TAA, tumor-associated antigen; TCR, T cell receptor.

The peripheral T_reg_ population, associated with suppressive functions [46], was found to be expanded in breast cancer patients compared with healthy individuals [15,52,64,71,72,73,74] (Figure 1, left). It was also correlated with other indicators of poor prognosis, like tumor stage [71] and elevated levels of circulating tumor cells (CTC) [74] and MDSC [71]. In HER2^+^ patients, therapy regimens containing trastuzumab for primary and metastatic patients were found to reduce the numbers of T_regs_ [15,73], which rise again only in relapsing patients [73]. When Wang et al. compared the peripheral and intratumoral populations of T_reg_, they identified a subpopulation of circulating T_regs_ characterized as FOXP3^hi^/CD45 RA^−^, with suppressive phenotype. This population was remarkably similar to the tumor infiltrating counterpart, including overlapping clonal diversity. These data strongly suggest that this is the T_reg_ population that infiltrates the TME [75]. Compared with other peripheral T_reg_ subsets, this type II T_reg_ population upregulated the immunosuppressive markers CD39, CTLA-4, TIGIT, and ICOS and responded better to TGF-β and IL-10, while the response to pro-inflammatory IL-4 and IFN-γ was hampered. This is also supported by the observation that type II T_regs_ were considerably expanded in breast cancer patients, compared with healthy women. Moreover, patients in which type II T_regs_ had a stronger suppressor phenotype (improved response to TGF-β and IL-10) displayed shorter relapse-free survival (RFS). In fact, in this cohort of 118 patients, type II T_reg_ suppressive activity was considered a strong indicator of relapse independently of other clinical features, reflecting disease stage (with suppressive activity increasing during disease progression).

Regarding the effector CD8^+^ T cells, there seems to be an imbalance in breast cancer patients towards higher proportions of more differentiated subsets, with signs of activation and possibly exhaustion [76]. This includes the metastatic setting [76], where lower percentages of naïve CD8^+^ and higher percentages of more differentiated CD8^+^ confer clinical benefit after treatment with high-dose paclitaxel [77]. However, breast cancer subtype can be determinant of this shift in T cell differentiation, given that HER2^−^ and hormone receptor-positive patients harbor a higher proportion of naïve and T_CM_ (central memory subset) than more mature CD8^+^ T cell populations [52,78], while the opposite is seen in HER2^+^ patients [52]. Nevertheless, circulating T cells appear to be at an earlier maturation stage compared with TIL [76], which may be an advantage for the application of immune-based therapies that rely on re-education of T lymphocytes. The memory phenotype of T cells also appears to be temporarily magnified by systemic chemotherapy, with memory and naïve populations returning to baseline only several months after the end of treatment [79].

Overall, there is abundant evidence of functionally deficient T cells in breast cancer patients [48,71,74,80,81,82,83,84,85] (Figure 1, left). Compared with those of healthy individuals, these T lymphocytes display a significant reduction in the secretion of pro-inflammatory cytokines IL-2, IFN-γ, IL-21, TNF-α, and IL-4 [48,74,80,81], with impaired responses to proliferative signals [48,82,83] as well as to IL-6 [71,85], IL-17 [71], and IFN-α and -γ [84]. Concomitantly, defects in several signal transducer and activator of transcription (STAT) signalling pathways (namely, STAT1, 3 and 5) were observed [71,82,84,85], indicating that patient T cells are likely less able to respond to a plethora of immune modulatory soluble factors [86,87]. These findings may have clinical significance, as it has been reported that increased CTC, a known indicator of poor prognosis, is associated with lower levels of IFN-γ, IL-2, and TNF-α secreted by T cells [74,80]. Further, a combination of a low CTC count and a high proportion of IFN-γ secreting CD8^+^ T cells improves OS [74]. Other studies have also highlighted the relationship between impaired IL-6 signalling and a heightened relapse rate [85], identifying baseline T cell response to IL-6 as a predictor of RFS in breast cancer [71]. Mego et al. reported that low levels of CD4^+^, IL-2^+^, CD8^+^, IFN-γ^+^, and TNF-α^+^ T cells posed a significant negative impact in patient survival [74]. Once again, this data points to the relevance of an effective, pro-inflammatory T cell response for better disease management.

T cell function may also be evaluated by the quality and magnitude of response to tumor-associated antigens. This becomes especially useful in the case of HER2^+^ tumors since HER2 is a well-characterized TAA [88]. In this regard, although overall CD8^+^ and γδ T cell cytotoxicity was found to be inferior to that of healthy individuals [81,82,89], the specific response to TAA was heightened [90], especially in HER2^+^ cases [52,88]. This response was found to correlate with improved survival and earlier tumor stage [91,92,93] (Figure 1, left). Similarly, patients who displayed a more diverse T cell receptor (TCR) repertoire and were able to maintain that diversity during chemotherapy (when CD4^+^ populations usually decline [94,95] and CD8^+^ increase [79]), were more likely to evolve positively and to harbor HER2^+^ tumors [96]. Remarkably, Bailur et al. found a clear association between CD8^+^ T cell response to HER2 and low levels of circulating MDSC, which conferred these patients a 100% survival rate at five years [91,92]. The production of granzyme B is one of the factors affecting the lytic capacity of T cells [89]. This is a protein that induces apoptotic cell death in the target cell. In line with this, a specific mutated genotype of the *GRZB* gene which leads to inferior cytotoxic function (48 R/R, 88 A/A, and 245 H/H) was found to confer a 16-times higher probability of developing breast cancer than the wild-type [89], emerging as a potential genetic biomarker for cancer susceptibility.

There is also evidence that peripheral T cells of breast cancer patients are more susceptible to apoptosis, as indicated by the expressive proportion of Fas^+^ AnnexinV^+^ lymphocytes in circulation [84,97,98] (Figure 1, left); these cells are more often CD25^−^ and occur in patients presenting a decrease in soluble Fas ligand (sFas-L) [97], suggesting that the pool of naïve and non-activated memory T cells is being selectively depleted by binding of sFas-L in circulation. T cell death may also be implicated in worse disease prognosis, as seen by the association between a higher number of CTC and elevated Fas^+^ T cells [97,98].

### 3.2. B Cells

The influence of B cells in the cancer promotion/regression axis is still largely understudied. B cells are antigen-presenting cells (APC), with the putative capacity to promote TAA-directed immune responses and the exclusive ability to produce antibodies, which may enhance the anti-tumor response [99]. However, most of the communication between B cells and the tumor contexture (not just locally but also systemically) remains unknown, such as: which factors and signalling pathways mediate the crosstalk between B cells and other immune effectors, how different stimuli may elicit a pro- or anti-inflammatory function in the TME, and the specific roles of each subpopulation (given that B cells exhibit high heterogeneity and plasticity), among others. Furthermore, it is essential to inquire about potential relationships between B cell response or other B cell-related biomarkers and disease prognosis that may be used to improve patients’ treatment in the clinical setting.

The available literature regarding circulating B cells in breast cancer patients is mostly recent and reports conflicting results on the proportion of B cells present in the peripheral blood [64,100,101,102]. While some authors claim that patients do not differ from healthy women in the number of circulating B cells [64,100], others present evidence that the amount of B cells is higher in breast cancer patients [101]. Additionally, the authors reported that increased circulating B cells raised the risk of developing breast cancer by 17% in otherwise healthy, pre-menopausal women [102]. The B cells of breast cancer patients were also found to be enriched for memory subsets [101] (Figure 1, middle), although this tendency was reversed after treatment with systemic chemotherapy [103]. On this note, both NAC and chemotherapy in the adjuvant setting were found to consistently deplete the pool of peripheral B cells [64,94,103,104,105]. The dynamics of depletion and repopulation were dependent on whether the patient received taxane-based therapy. Nonetheless, B cell numbers did not rise to their baseline level even nine months after treatment (contrary to other immune populations) [103]. In the metastatic setting, one cohort of 482 patients displayed a significant association between increased number of circulating CD19^+^ B cells and prolonged survival, although no connection was found between B cell counts and tumor subtype [106].

In recent years, a new subtype of B cells named B regulatory cells (B_regs_) has been brought to attention due to its role in enhancing a pro-tumorigenic environment [107,108]. These cells are highly heterogeneous in surface marker expression and, in fact, may not even constitute one bona fide B cell lineage but rather a functional state [107,109]. However, they were found to have in common the secretion of IL-10 and TGF-β, which renders them able to convert conventional CD4^+^ T cells into T_regs_, and thus subvert several immune effectors at the tumor site [110]. Knowing this, the finding that the population of circulating B_regs_ is reduced in breast cancer patients after systemic chemotherapy [103] (Figure 1, middle) may suggest that the post-chemotherapy period is a window of opportunity to introduce new immune-activating therapeutic options. This becomes especially relevant if we consider that tumor infiltrating B lymphocytes (B-TIL) have been positively associated with improved prognostic indices in TNBC [111] and highly proliferative breast carcinomas [112], indicating it may be possible to further amplify the beneficial anti-tumoral effects of this immune population [99].

### 3.3. NK Cells

Natural killer cells were first identified for their role in cancer immunosurveillance given their innate ability to selectively kill transformed cells without the need for previous cellular activation (contrasting with the other major killer of the lymphoid lineage, CD8^+^ CTLs) [113]. Unsurprisingly, NK cell number and phenotype have long been investigated for their potential use as cancer progression biomarkers and, more recently, strategies for enhancing NK function to improve anti-cancer therapies have become a new research trend in oncoimmunology [113,114,115]. As seen in other solid tumors [113], breast cancer patients typically display impaired NK cell function, albeit to varying degrees [50,54,55,64,81,84,116,117,118,119,120] (Figure 1, right). Although the proportion of circulating NK cells appears in the healthy range [15,64,119,120,121,122], they display a marked reduction in their ability to kill target tumor cells (either by direct cytotoxicity or by CD16-mediated ADCC) and to secrete pro-inflammatory cytokines [50,54,55,116,117,118,119,120]. A clear impairment in their sensitivity to stimulation [54] can also be seen by the decreased response to IFN type I [81,84] and downregulation of IL-2R [120] compared with healthy NK. Moreover, this diminished cytotoxic capacity was associated with indicators of cell death, such as AnnexinV positivity [120] and LDH leakage [55], and shown to deteriorate further with disease progression [54,55,123]. These data indicate that peripheral NK cells are being heavily influenced by the suppressive features of breast cancer. Concomitantly, disease progression promotes further downregulation of activating receptors (NKp30, NKG2D, DNAM1, and 2B4) and upregulation of inhibitory receptors (NKG2A and CD85j) [116], which was already apparent compared with healthy individuals [64,116,121]. NK cells from patients in advanced disease stages also exhibit a declining responsivity to cytokines [81,84], due in part to defects in STAT signalling pathways [54]. When compared with tumor infiltrating NK cells, their peripheral counterparts display a similar downregulation of activating receptors [116] despite maintaining higher functionality [116]. Still, this can be abrogated in presence of TNBC cells [123], tumor supernatant [116,122] or serum from metastatic patients [54]. Surprisingly, serum from early stage breast cancer patients seems to induce the opposite effect of improving NK function [54], suggesting that the level of systemic immune suppression in initial disease stages may still leave room for immune cell activation.

Consistent with the establishment of an immune suppressive environment at the systemic level, breast cancer progression is accompanied by a shift in NK cell subset distribution [124] (Figure 1, right), with an increase of cytokine-producing CD56^bright^ CD16^lo/−^ cells in circulation and a decrease in the predominant cytotoxic CD56^dim^ CD16^+^ subset [122], possibly due to a higher death rate of the latter [120]. This imbalance may negatively impact the pool of available effector killer cells, given that a higher proportion of CD16^+^ NK cells appears to correlate with more efficient ADCC [125]. The capacity of NK cells to perform ADCC becomes especially important in the context of HER2^+^ breast cancer treated with the monoclonal antibody trastuzumab [114]. For that reason, several studies have investigated potential predictors of ADCC efficiency and association with clinical response [125,126,127,128,129]. The CD16 (FcγRIII) and CD32 (FcγRII) receptors have received some attention due to their role in recognizing the Fc portion of trastuzumab (and other IgG antibodies), but while some groups claim that the genotype 158 V/V for CD16 confers higher ADCC efficiency [125,126], it appears the opposite may hold true for metastatic patients [126,127]. Reports for CD32 are also inconsistent, with some studies presenting a positive association between *FCGRIIA* 131 H/H genotype and trastuzumab clinical benefit [126,127], which was found to be non-significant in a larger cohort [128]. Nevertheless, the rate of ADCC itself may indicate which patients have the best likelihood of response to trastuzumab therapy, as seen in different cohorts of early stage [125,126] and metastatic patients [129]. This standard-of-care for HER2^+^ patients was also reported to improve NK cell cytotoxicity [125] (Figure 1, right), which was correlated with longer PFS in the metastatic setting [129]. Administration of chemotherapy regimens also appears to improve the effector phenotype of NK cells [50], especially in taxane-containing formulations [130], with upregulation of activating receptors [64] and higher production of cytotoxic granules [119]. Of note, women with large and locally advanced breast cancer who benefitted the most from neoadjuvant chemotherapy displayed significantly higher NK cytotoxicity at baseline [119], while another cohort of stage II–IV patients exhibited a positive association between increased NK cytotoxicity post-NAC and metastasis reduction [131]. These results highlight the potential use of NK effector function as biomarker for clinical response. This is further supported by the findings that NK cell function is impaired in families with higher incidence of breast cancer [132], and that former breast cancer patients (in remission for over five years) express NK activating receptors at a similar level as healthy individuals [116].

## 4. The Myeloid Lineage

Myeloid cell function and polarization are considered instrumental to the general immune response towards cancer in the human organism [133]. Tumor associated macrophages (TAM) are known promoters of metastization and cancer growth and comprise the major leukocyte population in breast cancer infiltrates [134]. However, other cell populations in the tumor milieu must also be acknowledged such as dendritic cells, for their capacity to capture tumor antigens and mount anti-tumor immune reactions [135], and neutrophils, which may become strongly polarized to act against or pro tumorigenesis [136]. The onset and development of breast cancer are also associated with subsets of myeloid-derived suppressor cells (MDSC), which are strongly immunosuppressive cells from the monocytic (m-MDSC) or granulocytic (g-MDSC) lineages. MDSC restrict immune effector function at the tumor site and promote recruitment and survival of T_regs_ [137]. Most importantly, it is known that part of these tumor-infiltrating populations is replenished from circulating precursors [134,136,137,138]. These data highlight the relevance of characterizing peripheral myeloid subsets to gain insight into the breast cancer–immune system crosstalk, as well as to procure biomarkers of disease progression.

### 4.1. Dendritic Cells

**Figure 2:**
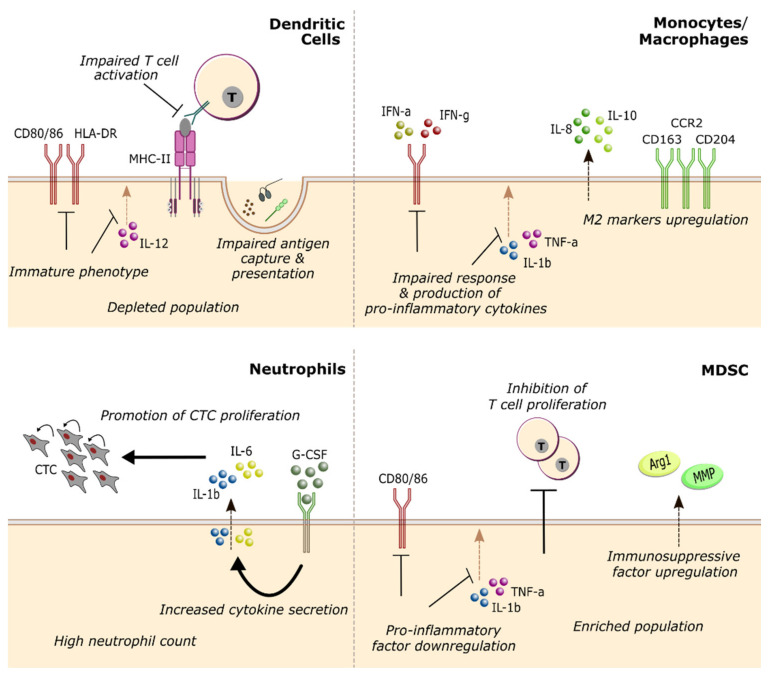
Surface markers and cytokine response of myeloid peripheral immune cell populations associated with breast cancer occurrence and/or progression. In the myeloid lineage, breast cancer patients display a marked increase in cell populations with pro-tumorigenic functions (like MDSC and neutrophils), while anti-tumorigenic populations of dendritic cells are depleted and the remaining dendritic cells (DC) acquire an immature phenotype, preventing the mounting of effective immune responses. Monocyte and macrophage function is also skewed towards immune suppression, with impaired response to pro-inflammatory stimuli and upregulation of M2 markers. CTC, circulating tumor cell; MDSC, myeloid-derived suppressor cell.

One of the key events in the establishment of anti-tumor immunity is the presentation of tumor antigens to different T cell subsets and their activation [135]. Although other immune populations (like B cells and macrophages) can also present antigen, dendritic cells are considered professional antigen presenting cells due to their ability to induce a primary immune response in naïve T cells [135]. However, the pivotal role of DC in cancer immunity has two distinct sides, since DC function can also be exploited by the tumor to inhibit immune effectors in the TME, either via checkpoint ligands like PD-1 or soluble factors like IL-10 [138]. In breast cancer patients, circulatory DC also appear to be negatively influenced by the cancer-driven systemic immune suppression. In breast cancer patients, DCs have been described to present impaired function [48,83,139,140,141,142] and reduced numbers [139,143] compared with healthy individuals (Figure 2, up left). This decrease in the DC population is also indicative of disease progression [83,144], as a continued decline of the two major DC subsets has been described in more advanced cancer stages, namely, with conventional DC (cDC) [140,144] and plasmacytoid DC (pDC) [92,140,144].

Overall, circulating DC appear to be progressively enriched in immature cells [140,144] and to exhibit marked defects in phagocytosis, antigen capture and presentation [48,140,144], and T cell activation [48,83,141,144] (Figure 2, up left). On the other hand, an earlier study with 53 patients observed an increase in mature CD83^+^ DC from stage I to stage II breast cancer that was abolished upon tumor resection [139]. These results suggest that the primary tumor may also activate dendritic cells in earlier stages. Phenotypically, patients’ DC display impaired IL-12 production upon stimulation [139,141,142] as well as downregulation of HLA-DR [83,141,144], CD80, and CD86 [48,83,140,141,144], which are essential for antigen presentation and T cell activation [135]. Poor cytokine secretion by cDC was also significantly associated with elevated numbers of CTCs in patient blood [142], further suggesting a progressive decline in DC function. In the few studies where prognostic parameters have been assessed, patients with higher levels of circulating DC post-treatment consistently showed longer survival [77,92], despite the depletion of the DC pool induced by chemotherapy [144]. Nevertheless, there is still a great need for studies clarifying the association between dendritic cells (numbers, subsets, and phenotypes) and the clinical prognosis of breast cancer patients—more so, if one considers the abundance of cancer immunotherapies under development that depend on DC activity [135,138].

### 4.2. Monocytes and Neutrophils

Monocytes and the monocytic lineage (including macrophages and other subpopulations) have long been associated with breast cancer outcome and response to treatment, considering the role of tumor-associated macrophages in cancer progression [134]. On the other hand, the phenotype and function of circulating monocytes have not been under the same spotlight. Nevertheless, accumulating evidence starts to indicate a prevalence of anti-inflammatory polarization of peripheral monocytes in breast cancer patients [81,85,145,146], akin to what can be found at the tumor site [134]. Peripheral blood monocytes from patients display a decreased responsiveness to IFN-γ [85] and IFN-α [81], and impaired production of pro-inflammatory cytokines [145] compared with healthy individuals. Simultaneously, the secretion of anti-inflammatory IL-8 and IL-10 is increased [145] (Figure 2, up right). Breast cancer patients were also found to carry a higher proportion of M2-like macrophages in circulation [146]. A decline in monocytic pro-inflammatory response is also a sign of disease progression [85] which correlates with poor outcome parameters (e.g., TAM infiltration, CSF-1R upregulation, and shorter RFS in early-stage patients) [85]. Coincidentally, metastatic patients with more M2-like [147] and fewer HLA-DR^+^ mature monocytes [77] in circulation also presented worse prognostic indicators and survival times, respectively. However, it is interesting to note that one cohort of relapsing patients with metastasis displayed an increase in responsiveness to IFN-α and IL-12 production [81]. Overall, these data advance the possibility of using peripheral monocyte phenotype as a prognostic marker through several disease stages, but also prompts the need for further research on the mechanisms behind their pro- or anti-inflammatory polarization. Moreover, in luminal A breast cancer patients, advanced disease was associated with a shift towards higher representation of intermediate (CD14^+^ CD16 ^+^) over classical (CD14^hi^ CD16^−^) monocytes [104], reflecting the lack of immunosuppression associated with this subtype [69].

The predictive potential of monocyte count remains controversial. While some studies indicate an association between high peripheral monocyte counts and increased CD8^+^ TIL (a positive prognostic indicator) [30], or lower risk of developing breast cancer [102], other cohorts displayed a clear clinical benefit from lower monocyte counts, either due to fewer CTC in blood [36] or prolonged disease-free survival [31,37,77]. Work by Lafrenie et al. also alerts to the distinct prognostic values that can be obtained from monocyte proportions in the peripheral blood depending on the method for calculating such proportions and on patient treatment regimen [77].

On a similar note, the implications of circulating neutrophils for breast cancer have only recently been considered. Still, the available literature has been quite consistent in showing that high neutrophil counts are predictive of poor prognosis for breast cancer patients, associated with higher levels of circulating tumor cells [36] (Figure 2, down left), lymph node invasion [28] and drastically inferior survival probabilities [28,32]. Szczerba et al. describe a close relationship between neutrophil phenotype and promotion of metastasis in a cohort of invasive breast cancer patients [148]. They observe the capacity of CTC-associated neutrophils to induce CTC proliferation via IL-6 and IL-1β and alert that G-CSF containing therapeutics may inadvertently promote an increase in CTC-neutrophil clusters [148].

### 4.3. MDSC

In breast cancer patients, both MDSC subsets (m-MDSC, CD14^+^ HLA-DR^−^; and g-MDSC, CD14^−^ CD11b^+^ CD15^+^ [137]) appear to be more abundant than in healthy individuals [64,71,78,145,149,150,151]. These MDSC display typical indicators of suppression: inhibition of T cell proliferation [78,149]; downregulation of CD80/86, TNF-α, and IL-1β [145]; upregulation of matrix metalloproteinases and arginase (*ARG1*) [145] (Figure 2, down right). Increased numbers of MDSC were found to be associated with advanced tumor stage [71,149,151], higher metastatic burden [145,151] and high T_reg_ frequency [145], all predictors of poor prognosis. The decrease in MDSC after surgical resection of the tumor [150] suggests a preponderant role of tumor-secreted factors in maintaining this immune population. The response of each MDSC subset to systemic chemotherapy appears to be distinct, with an increase of g-MDSC [151,152] and a decline in m-MDSC [153]. Nevertheless, lower levels of g-MDSC at baseline [154] and after NAC [152] were more frequent in patients achieving pCR after this therapy. The predictive power of MDSC count may be more relevant when considered together with another parameter of immune function; in a cohort of elderly breast cancer patients, the presence of reduced levels of m-MDSC and a positive CD8^+^ T cell response to TAA conferred a 100% probability of survival at 4 years [91].

## 5. Experimental Models to Interrogate Tumor–Immune Interactions

It is well established that the peripheral immune landscape of breast cancer is characterized by complex cell states and phenotypes, and that some of these populations display strong correlation with disease prognosis and therapeutic outcomes. Nonetheless, current knowledge is not enough to understand the biological interactions between immune populations and tumor cells and how these interactions are modulated by different pharmacological approaches. This knowledge is instrumental to unravel new biomarkers, as well as to define the weak spots of cancer immunosuppression and identify the best effector functions to be exploited for each immune subset. This knowledge can leverage the development of novel immunotherapies and a refined assignment of current standard-of-care regimens to breast cancer patients.

During the last decade, it became increasingly clear that to obtain accurate predictors of drug response it was necessary to develop new experimental cancer models taking into account: the native tri-dimensional (3D) tumor architecture, the cellular composition of the tumor microenvironment, and the human relevance of the underlying interactions [155,156,157,158,159]. Despite the fundamental role of mouse models in many areas of immunology research, it is clear that they are unable to serve as faithful replicas of the human immune system or human tumors [159,160,161], also rendering them less reliable for the testing of immunomodulatory drugs or therapeutic proteins [159,160]. This is in part due to particular differences in the composition and function of both immune systems, some well documented such as NK receptor and major histocompatibility complex (MHC) repertoires or T cell subsets, and potentially others that are still unknown [160]. Further, mice strains lack genetic diversity and exposure to pathogens, and they have a short life span. Altogether, these features contribute to the compromised ability of the murine immune system to generate the variety of processes and cell states observed in the human [161]. Furthermore, the alarmingly high rate of drug failure when transitioning to clinical trials has been pointing to the poor transition of pre-clinical mouse models [162,163]. The use of humanized mice as an alternative to study human immune effects at the system level also falls short of the ideal [159,164,165]. Mice transgenic for human proteins still carry a murine immune system [159], and even the strains used for engraftment of human hematopoietic stem cells do not reflect the full diversity and function of the human immune system [159,164]. Additionally, animal-based research is always more expensive and time consuming, and fails to take into consideration the role of human stromal and endothelial cells [165], which are known to be closely involved in the tumor–immune crosstalk [157].

In this context, 3D heterotypic cellular models of tumor–immune interaction with a human-only cellular composition have surfaced as a valuable alternative to the overly simplistic 2D cancer and immune cell lines-based research and to the often inadequate and more expensive animal-based in vivo models [166,167]. Broadly speaking, to grasp the dynamics of tumor–immune interactions it would be critical to model two key biological contexts: (a) the TME characteristic of each breast cancer subtype, often exerting strong immunosuppression over its immune component; (b) the contact between peripheral immune cells and the already developed TME. While in the first the goal is to either eliminate or repolarize suppressive cells, in the second there is an added clinical opportunity to use the pro-inflammatory potential of immune cells which have not yet been subverted by the tumor. Both these approaches can be used to establish platforms for three areas in which clinical translation remains ineffective: drug testing, biomarker discovery and biomarker validation [168,169]. While patient-derived explants (PDE) have already been used to identify prognostic [170] and predictive [171] biomarkers in solid tumors, other advanced 3D cellular models incorporating key features of the tumor–immune interaction can be essential to identify and validate peripheral immune biomarkers of breast cancer [172,173,174].

At this point, it should be noted that immunocompetent breast cancer 3D models are scarce in the literature, and that existing ones are mostly proof-of-concept studies that have not been replicated or used for further testing (Table 2). Currently available models of the breast cancer microenvironment often incorporate particular immune subpopulations, such as monocytes [175,176,177,178], T cells [179,180], or NK cells [180,181], to capture the reciprocal influence of immune and tumor cells in the suppressive environment [175,176,177,178,181] but also to assess the anti-tumor capacity of killer cells [179,180]. In another approach, several groups have developed models containing distinct immune cell types for drug testing in the TME of different breast cancer subtypes [182,183]. These studies heighten the promise of advanced heterotypic cell models for predictive pre-clinical and co-clinical studies. On the field of patient-derived ex vivo cultures (explants [172] and precision cut slices [184,185]), fewer examples can be found due to the difficulty in maintaining the native immune component, as well as the stroma of the breast cancer TME in prolonged culture [172], similar to what is observed in other cancer types [186]. Our group has recently described a culture method (using alginate-encapsulated tumor tissue from ER^+^ breast cancer patients, cultured in an agitation-based system) in which expression and functionality of ER receptors are retained after four weeks, as well its CD45^+^ population [187]. This work highlights the potential of ex vivo cultures to study the temporal dynamics of native TME interactions, although further characterization is necessary to probe putative drug testing applications.

Confrontational 3D models have most often been used to study immune cell recruitment and initial infiltration into the tumor mass [188,189,190,191,192,193], investigating the underlying tumor- and stromal-derived soluble factor-induced signalling [191,192,193]. Alternatively, other models of immune cell contact with the tumor mass have assessed means to activate NK cells [189,190], CTL [194], and monocytes [194] towards tumor cell killing. Work by Wallstabe et al. displayed the proof-of-concept that immunocompetent breast cancer models can be valuable for testing the killing efficacy of CAR-T cells in the pre-clinical setting, while also taking into account T cell extravasation from the tumor vasculature [195].

Nonetheless, these models still reflect a much lower complexity compared to animal models, the current gold standard of mechanistic studies, pre-clinical testing, and biomarker discovery. Moving towards a research paradigm mostly based on human-only systems requires substantial technological advancements in the area of human immune modelling—ideally leading to the development of an “artificial immune system” built from modular units, each representing the function of an essential immune organ or compartment [165,196]. As of today, there are several reports on the development of advanced cellular models for lymph nodes [197], spleen [198], bone marrow [199], tonsil [161], thymus [200], and immunocompetent skin [201], lung [202], gut [203], and liver [204], albeit with varying levels of functionality and native-like architecture. The generation of immune organ-like structures in vitro has been hampered by difficulties in recreating these very complex microenvironments, with uncommonly high cell densities and defined functional zones, on a small scale, and specific dynamics of cellular differentiation, communication and motility [165,205]. The field of microphysiological systems (MPS) (also known as “organs-on-chip”) combined with advancements on bioprinting can be instrumental in the development of novel advanced models that more closely resemble human tumor–immune interactions, and that allow the modular integration of several immune “organs” or compartments [165,196,206]. At the moment, the main challenge of this technology is the implementation of sensors for live monitoring of cellular processes, which can be based on the miniaturization of existing analytical techniques or in the integration of novel biosensors [207]. By bringing together the potential for broad customization of culture conditions, the advantage of miniaturization and throughput (compared with traditional bioreactors or culture plates), and the possibility of replicating different biological compartments of the TME in the same system [208,209], MPS technology may be a starting platform to refocus on basic and applied human immunology research [165,196].

**Table 2:** 3D cellular models of breast cancer and immune system interaction.

Model Type	Immune Cell Types	Culture Time	Model Objective	Major Observations	Refs.
***Tumor Microenvironment Models***					
Spheroid-based	Monocytes (+ stroma)	7 days	MΦ polarization in the TME	TNBC TME induces stronger M2-like MΦ polarization including secretion of pro-tumoral cytokines and MMPs	[175]
Organoid	T cells	6 h	T cell killing	Vδ2+ T cells effectively kill BC cells in response to bisphosphonate drugs	[179]
Spheroid-based	T cells, NK cells	4 days	Tumor interaction with Treg and NK cells	Immune mediation affects morphology of the tumor mass and secretion of CCL4	[180]
Spheroid-based	Monocytes	7 days	MΦ-induced angiogenesis	MΦ induce increasing VEGF production in the TME over time	[176]
Spheroid-based	Monocytes	5 days	MΦ polarization in the TME	Aggressiveness of BC subtype correlates with upregulation of MMP1/9 and COX2, collagen degradation and production of PGE2	[177]
Spheroid-based	Monocytes	7 days	Monocyte differentiation in the TME	Monocytes in the TME may have the potential to differentiate into endothelial cells	[178]
Spheroid-based	NK cells	2 days	Tumor escape from NK surveillance	Tumor exposure induces a transcriptional “resting” state in NK cells that promotes tumor growth	[181]
Spheroid-based	CD45^+^ (+ stroma)	10 days	Drug testing in ER^+^ TME	Inhibition of PDGF and IL-1 signalling synergizes with tamoxifen treatment in ER^+^ BC	[182]
MPS	PBMC (+ stroma)	4 days	Drug testing in HER2^+^ TME	Long-term cancer-immune interactions and ADCC induced by trastuzumab treatment are counteracted by cancer-associated fibroblasts	[183]
PDE	CD45^+^ (+ stroma)	4 weeks	Maintenance of ER^+^ TME	CD45+ cells can be maintained in a long-term culture of patient-derived explants	[187]
Precision-cut slices	CD45^+^ (+ stroma)	1 day	Drug testing in the TME	Rapamycin modulates expression of several genes associated with biosynthetic and catabolic processes in HER2^+^ and HER2^−^ BC	[185]
***Peripheral immunity—TME Confrontational Models***					
MPS	Monocytes, T cells (+ endothelial)	6 days	T cell recruitment	T cell recruitment to the tumor site is promoted by a hypoxic TME containing monocytes	[188]
MPS	NK cells (+ endothelial)	3 days	NK cell recruitment, infiltration, and cytotoxicity	NK cells actively migrate and infiltrate the tumor mass and respond to antibody-cytokine conjugates with enhanced cytotoxicity	[189]
Spheroid-based	NK cells	2 days	NK recruitment and infiltration	Bispecific CD16/mesothelin antibody promotes NK cell recruitment, infiltration, and dose dependent ADCC	[190]
Spheroid-based	Macrophages	2 days	Monocyte migration and tumor invasion, tumor-immune communication	Tumor-secreted miR-375 enhances MΦ migration, infiltration and pro-tumoral phenotype	[191]
Spheroid-based	Monocytes (+ stroma)	40 h	Monocyte migration and invasion	Monocyte migration and invasion capacity depends on BC subtype and is promoted by presence of fibroblasts partly via CCL2 signalling	[192]
Spheroid-based	Monocytes	2 days	Monocyte recruitment and invasion	Increased ROS production upon disruption of mammary epithelium polarization enhances monocyte recruitment and infiltration	[193]
Spheroid-based	PBMC	2 days	Initial anti-tumor immune response	CD80 expression on phagocytes is required to induce CTL activation and is negatively regulated by PGE2	[194]
MPS	T cells	3 days	Test anti-tumor CAR T function	ROR1-CAR T cells actively migrate from the periphery, infiltrate, and eliminate several layers of the tumor mass	[195]

ADCC, antibody-dependent cell-mediated cytotoxicity; BC, breast cancer; CTL, cytotoxic T lymphocyte; ER, estrogen receptor; MΦ, macrophage; MPS, microphysiological system; PBMC, peripheral blood mononuclear cells; PDE, patient-derived explant; TME, tumor microenvironment; TNBC, triple negative breast cancer.

## 6. Conclusions and Future Perspectives

The current burden of breast cancer for women worldwide requires new and better measures for disease management, monitoring, and treatment tailoring. Predictive and prognostic immune biomarkers obtained from the peripheral blood would certainly make for faster, cheaper, and more individualized decision making, but as of today much of the relationship between the peripheral immune system and breast cancer is not uncovered. In this review, we have summarized the available knowledge of the peripheral immune landscape of breast cancer but, as it became clear, this is a field that still requires attentive interrogation and standardization to generate meaningful clinical information. Currently, only a limited number of immune features have shown plausible association with clinical data for breast cancer. These include the marked relationship of neutrophils with poor prognosis across subtypes (Section 2.1), as well as the link between more reactive cytotoxic lymphocytes (CD8^+^ T cells and NK cells) and better outcome for HER2^+^ breast cancer patients (Section 3.1 and Section 3.3, respectively). However, these indicators still require thorough research and validation before being introduced as prognostic biomarkers in clinical practice. In future studies, attention must be paid to evaluate separately patients from different breast cancer subtypes, as these have been shown to display distinct immune profiles. An important caveat to consider is that findings from profiling of small patient samples require further validation in larger patient cohorts. The identification of clinically relevant peripheral immune biomarkers will also boost the development of advanced cellular models of cancer–immune interaction, much needed to uncover molecular mechanisms and putative drug targets in an all-human setting. Despite major advancements made in recent years, including the rise in popularity of “organs/tumors-on-chip”, immunocompetent tumor models still face two considerable challenges: the need to incorporate tissue-specific stroma (which may be easier for a cancer environment, but more difficult for, e.g., lymph node models) and the requirement for functional vascularization, allowing for immune cell perfusion and communication with functional endothelial cells. Above all, it is essential to keep in mind that improvements in advanced models of human immunity need to be preceded by a new look on basic human immunology, one that also includes extensive patient-based research for biomarkers of interest.

## Figures and Tables

**Table 1:** Clinical findings associated with peripheral lymphocyte counts.

Patient Cohort	Disease Stage	Cohort Size	Prognostic/Predictive	Major Observations	Refs.
***Peripheral Blood Lymphocyte Count***					
Not stratified	Primary BC	103	Both	Low PBL associated with short DFS, increased metastization and progression after NAC treatment	[27]
Not stratified	Primary BC	180	Predictive	High PBL improves likelihood of pCR after NAC	[28]
Not stratified	Primary BC	145	Prognostic	High PBL associated with higher TIL infiltration	[30]
Not stratified	All	305	Prognostic	High PBL associated with early disease stages and no metastization	[26]
>65 years old	All	69	Prognostic	High PBL associated with longer DFS at 3 years	[29]
HR+	Primary BC	Unknown	Prognostic	High PBL associated with longer OS and DFS	[30]
HER2+	Primary BC	Unknown	Prognostic	No prognostic association	[30]
TNBC	Primary BC	230	Prognostic	High PBL associated with longer OS and DFS	[31]
***Neutrophil-to-Lymphocyte Ratio***					
Not stratified	Primary BC	180	Both	Low NLR improves likelihood of pCR after NAC; high neutrophil count associated with shorter DFS	[28]
Not stratified	Primary BC	145	Predictive	Low NLR associated with increased probability of pCR after NAC	[30]
Not stratified	Primary BC	150	Both	Low NLR associated with longer DFS and OS, and lower risk of relapse after NAC	[32]
Not stratified	All	316	Prognostic	High NLR associated with increased short- and long-term mortality	[33]
Not stratified	All	437	Prognostic	High NLR associated with increased mortality at 5 years	[34]
Not stratified	All	1435	Prognostic	High NLR associated with higher metastization, HER2 positivity, HR negativity and mortality risk	[35]
Not stratified	Metastatic BC	516	Prognostic	Low NLR associated with shorter OS	[36]
TNBC, >65 years old	All	25	Prognostic	Low NLR associated with longer DFS and OS	[29]
>65 years old	All	113	Predictive	Low NLR associated with increased probability of pCR after NAC	[29]
***Lymphocyte-to-Monocyte Ratio***					
Not stratified	Primary BC	145	Prognostic	High LMR associated with longer DFS and OS	[30]
Not stratified	Primary BC	145	Prognostic	High LMR associated with higher TIL infiltration	[30]
Not stratified	Primary BC	150	Both	High LMR associated with longer DFS and OS and lower risk of relapse after NAC	[32]
Not stratified	Primary BC	542	Both	High LMR associated with HR positivity, longer DFS and improved response to NAC	[37]
Not stratified	Metastatic BC	516	Prognostic	High LMR associated with longer OS	[36]
>65 years old	All	69	Prognostic	No prognostic association	[29]
TNBC	Primary BC	230	Prognostic	High LMR associated with less advanced disease	[31]
TNBC	Primary BC	230	Prognostic	High LMR associated with longer DFS and OS	[31]
HER2^+^, TNBC	Metastatic BC	100; 124	Prognostic	High LMR associated with longer OS	[36]
Luminal	All	259	Prognostic	High LMR associated with longer DFS	[38]
***Platelet-to-Lymphocyte Ratio***					
Not stratified	Primary BC	145	Prognostic	No prognostic association	[30]
Not stratified	All	437	Prognostic	High PLR associated with increased tumor dimension, metastization, 5-years mortality rate and higher NLR, more likely to be HER2^+^	[34]
Not stratified	All	1435	Prognostic	High PLR associated with increased tumor dimension, metastization, 5-years mortality rate and higher NLR, more likely to be HER2^+^	[35]
Not stratified	Metastatic BC	516	Prognostic	Low PLR associated with shorter OS	[36]
>65 years old	All	69	Prognostic	No prognostic association (multivariate analysis); low PLR associated with longer DFS for TNBC	[29]
HER2+	Metastatic BC	100	Prognostic	Low PLR associated with shorter OS	[36]
Luminal B, Basal	Primary BC	251; 70	Prognostic	High PLR associated with shorter OS and metastization	[39]

BC: breast cancer; DFS: disease-free survival; HR: hormone receptor; LMR: lymphocyte-to-monocyte ratio; NAC: neoadjuvant chemotherapy; NLR: neutrophil-to-lymphocyte ratio; OS: overall survival; PBL: peripheral blood lymphocytes; pCR: pathological complete response; PLR: platelet-to-lymphocyte ratio; TIL: tumor-infiltrating lymphocytes; TNBC: triple negative breast cancer. “Not stratified” indicates that the observations were made in cohorts that may contain patients of any BC subtype and age.

## Abbreviations

ADCC	antibody-dependent cell-mediated cytotoxicity
APC	antigen-presenting cell
BC	breast cancer
BReg	B regulatory cell
CAR-T	chimeric antigen receptor T cell
cDC	conventional dendritic cell
CTC	circulating tumor cells
CTL	cytotoxic T lymphocyte
DC	dendritic cell
DFS	disease-free survival
ER	estrogen receptor
g-MDSC	granulocytic myeloid-derived suppressor cell
HR	hormone receptor
LDH	lactate dehydrogenase
LMR	lymphocyte-to-monocyte ratio
MΦ	macrophage
MDSC	myeloid-derived suppressor cell
MHC	Major histocompatibility complex
m-MDSC	monocytic myeloid-derived suppressor cell
MPS	microphysiological system
NAC	neoadjuvant chemotherapy
NLR	neutrophil-to-lymphocyte ratio
NK	Natural Killer cells
OS	overall survival
PBL	peripheral blood lymphocytes
PBMC	peripheral blood mononuclear cells
pCR	pathological complete response
pDC	plasmacytoid dendritic cell
PDE	patient-derived explants
PLR	platelet-to-lymphocyte ratio
PMA	phorbol myristate acetate
PR	progesterone receptor
RFS	relapse-free survival
ROC	receiver operator characteristic
STAT	Signal transducer and activator of transcription
TAA	tumor-associated antigen
TAM	tumor-associated macrophage
TCM	central memory T cell
TCR	T cell receptor
T-DM1	trastuzumab emtansine
TIL	tumor-infiltrating lymphocytes
TME	tumor microenvironment
TNBC	triple negative breast cancer
TReg	T regulatory cells
